# Willingness to vaccinate and willingness to pay for vaccination against peste des petits ruminants in northern Senegal

**DOI:** 10.3389/fvets.2024.1405073

**Published:** 2024-06-26

**Authors:** Guy Sidwatta Ilboudo, Francis Wanyoike, Sirak Bahta, Samba Sy, Cheikh Ahmed Tidiane Djigo, Papa Anoune Sall, Mamadou Moustapha Lô, Michel Dione

**Affiliations:** ^1^International Livestock Research Institute, Ouagadougou, Burkina Faso; ^2^International Livestock Research Institute, Nairobi, Kenya; ^3^Institut Sénégalais de Recherches Agricole/Bureau d'Analyse Macro-Economique, Dakar, Senegal; ^4^Agronomes et Vétérinaires Sans Frontières, Linguère, Senegal; ^5^Service Départemental de l’Elevage et des Productions Animales de Linguère, Linguère, Senegal; ^6^Laboratoire National de l’Elevage et de Recherches Vétérinaires, Dakar, Senegal; ^7^International Livestock Research Institute, Dakar, Senegal

**Keywords:** sheep, goat, vaccination, choice experiment, Senegal

## Abstract

**Background:**

This study was carried out in Linguere department, Louga region of Senegal. Its objective was to explore the socio-economic factors that influence small ruminant producers’ decisions on vaccination against peste des petits ruminants.

**Methods:**

We carried out a willingness to vaccinate and willingness to pay for vaccination using a choice experiment approach with 200 small ruminant producers.

**Results:**

Results showed that the key factors that influence willingness to vaccinate, include perceived benefits of vaccination (98, 95%CI: 96–100%), the type of vaccinator (91, 95%CI: 87–95%), the access to information (86, 95%CI: 81–91%), the vaccine availability (80, 95%CI: 74–86%), and the possession of a vaccination certificate by the producer (76, 95%CI: 70–82%). Preferences of producers leaned toward home vaccination (preference weight = 0.74, *p* = 1%), individual herd vaccination (preference weight = 0.45, *p* = 1%), elective participation to vaccination (preference weight = 0.33, *p* = 0.01), and low-cost services (preference weight = −0.004, *p* = 0.1). Producers expressed a willingness to pay for vaccination per animal of XOF 184 (USD 0.3), XOF 113 (USD 0.18), and XOF 82 (USD 0.13) for home, individual herd, and elective vaccination, respectively.

**Conclusion:**

The findings underscore the importance of targeted awareness campaigns and bringing vaccination services closer to the producers.

## 1 Introduction

Small ruminants (SR) - sheep and goats play a crucial role in the livelihood of livestock producers, particularly in developing countries like Senegal. With more than seven million sheep and six million goats in 2019, SR contribute significantly to the economy, society, and culture of agropastoral and pastoral communities in Senegal. However, the productivity of SRs is hampered by numerous challenges, including poor health management. Peste des petits ruminants (PPR) stands out as one of the deadliest diseases of SRs (1, 2).

Since reported in Senegal in 1955, PPR has become a significant obstacle to the development of SR production, causing severe disruptions to the livelihoods of affected producers (1, 3). In response to this challenge, Senegal established a compulsory vaccination campaign in 2002 through a public-private partnership. The estimated cost of vaccination is XOF 106 (USD 0.17) per vaccinated animal, with the government subsidizing up to 56 XOF (USD 0.09) and producers contributing XOF 50 (USD 0.08). Since 2018, as part of the global PPR eradication program led by WOAH (World Organization for Animal Health) and the FAO (Food and Agriculture Organization of the United Nations), the government of Senegal has organized mass vaccination campaigns, with a main of reaching over 80% post-vaccination coverage (1, 4). This initiative aims to significantly improve vaccination coverage to control the spread of PPR among SR populations through the establishment of herd immunity.

Despite significant efforts in terms of logistics for vaccine acquisition, distribution and vaccination cost subsidies, the post-vaccination coverage rate in most countries of the West African sub-region involved in the global PPR eradication strategy remains low in relation to the eradication objectives set for 2027 to 2030 (1). In Senegal, coverage remained below 25% until 2017 (1, 5).

Various studies have consistently reported numerous challenges related to SR vaccination in Senegal, including difficulties in mobilizing producers (1, 5, 6). Limited research has been conducted to understand producers’ opinions about the willingness to vaccinate (WTV) and the willingness to pay (WTP) for Vaccination against PPR.

The WTV and the WTP for vaccination depend on the level of information producers have about vaccination campaigns and their market orientation (6). For instance, surveys conducted in Mali have revealed that while some producers lack the means to vaccinate their animals, others perceive insufficient benefits to justify investing in vaccination. Additionally, some stakeholders argue that the limited participation of producers in vaccination is not solely due to the perceived high cost of vaccination but is also influenced by insufficient access to good quality vaccines and a lack of awareness during vaccination campaigns (6, 7). The access and utilization of vaccines by livestock keepers are governed not only by technical and logistical constraints but also by key social and cultural factors, preferences, and norms, particularly those related to gender (8).

Therefore, our study aimed to identify socio-economic factors that influence producer’ decisions to vaccinate their SRs against PPR. We also aim to describe the choices and preferences of producers regarding different vaccine attributes and vaccination services based on the local context. Ultimately, the study defined an acceptable premium price for PPR vaccination considering various attributes of vaccination, including the level of thermotolerance of the vaccine.

The results of this study are expected to equip disease control decision-makers with valuable information to make the right choices when developing livestock vaccination strategies.

## 2 Materials and method

### 2.1 Choice experiment

There are two primary approaches to measuring individuals’ WTP for a particular good: the revealed preference and stated preference approaches. The revealed preference approach, initially introduced by Samuelson, involves observing market behavior to gain insights into individual preferences (9, 10). Consumer preferences are revealed through their purchasing decisions under varying incomes and prices. This method relies on empirical choices derived from market data and different types of experiments (10). On the other hand, the stated preference approach assesses the value of individual attributes within a bundled good by collecting individuals’ stated preferences in hypothetical scenarios (11). This can be done through methods such as contingent valuation or conjoint analysis. Contingent valuation involves directly asking individuals about their WTP for a specific health activity, but it does not allow for the identification of the specific attributes on which individuals base their WTP (12). Conjoint analysis is a stated-preference survey method that enables the elicitation of responses that reveal preferences, priorities, and the relative importance of various features associated with healthcare interventions or services (11). Currently, these two methods are considered competitors for estimating WTP, and their respective performance is widely debated in the international literature (13).

Conjoint analysis offers several advantages over other methods. One advantage is that it explicitly presents respondents with substitutes, encouraging them to explore their preferences and trade-offs in more detail. This allows respondents to express ambivalence or indifference directly, resulting in relatively less non-response and protest behavior compared to other approaches (14). Additionally, incorporating respondent uncertainty can have a significant impact on the WTP among those who do respond. The most widely used form of conjoint analysis in health economics, outcomes research, and health services research is the discrete choice experiment (DCE). In a DCE, researchers control attribute levels experimentally and ask respondents to make choices among sets of profiles in a series of choice questions. This approach enables researchers to effectively reverse-engineer choices and quantify the impact of attribute level changes on decision-making. By using DCE, researchers can gain insights into preferences for changes in attributes and attribute levels (15).

The choice experiment conducted in this study adopts a multi-attribute stated preference approach, which allows for the assessment of the value of individual attributes within a bundled good, specifically a vaccine. By using individuals’ stated preferences in a hypothetical scenario (11), this method directly measures preferences and relates them to utility, enabling the estimation of economic values for vaccine attributes and willingness to pay for different vaccine options. The theoretical foundation of this framework draws upon the Lancasterian consumer theory and discrete choice random utility theory (16). To construct the choice sets, the vaccine attributes and their levels are identified and combined according to an experimental design, resulting in sets of discrete choice alternatives. Respondents are presented with a series of these choice alternatives and asked to indicate their preferred option. Each choice alternative is characterized by multiple attributes, including a monetary attribute that varies across alternative fields (11). By analyzing how respondents’ choices change as the attributes and monetary amounts are varied, analysts can gain insights into the relative importance of different attributes and assess their impact on decision-making. In this study, we conducted a choice experiment to assess producers preferences for vaccine attributes and their willingness to pay for different vaccination options.

### 2.2 Study area and sample size

The study was conducted in the Linguere department, located in the Louga region of Senegal. Linguere is the one of departments of the region where various partners implement interventions related to livestock production and PPR control (5, 17, 18).

**Figure 1:**
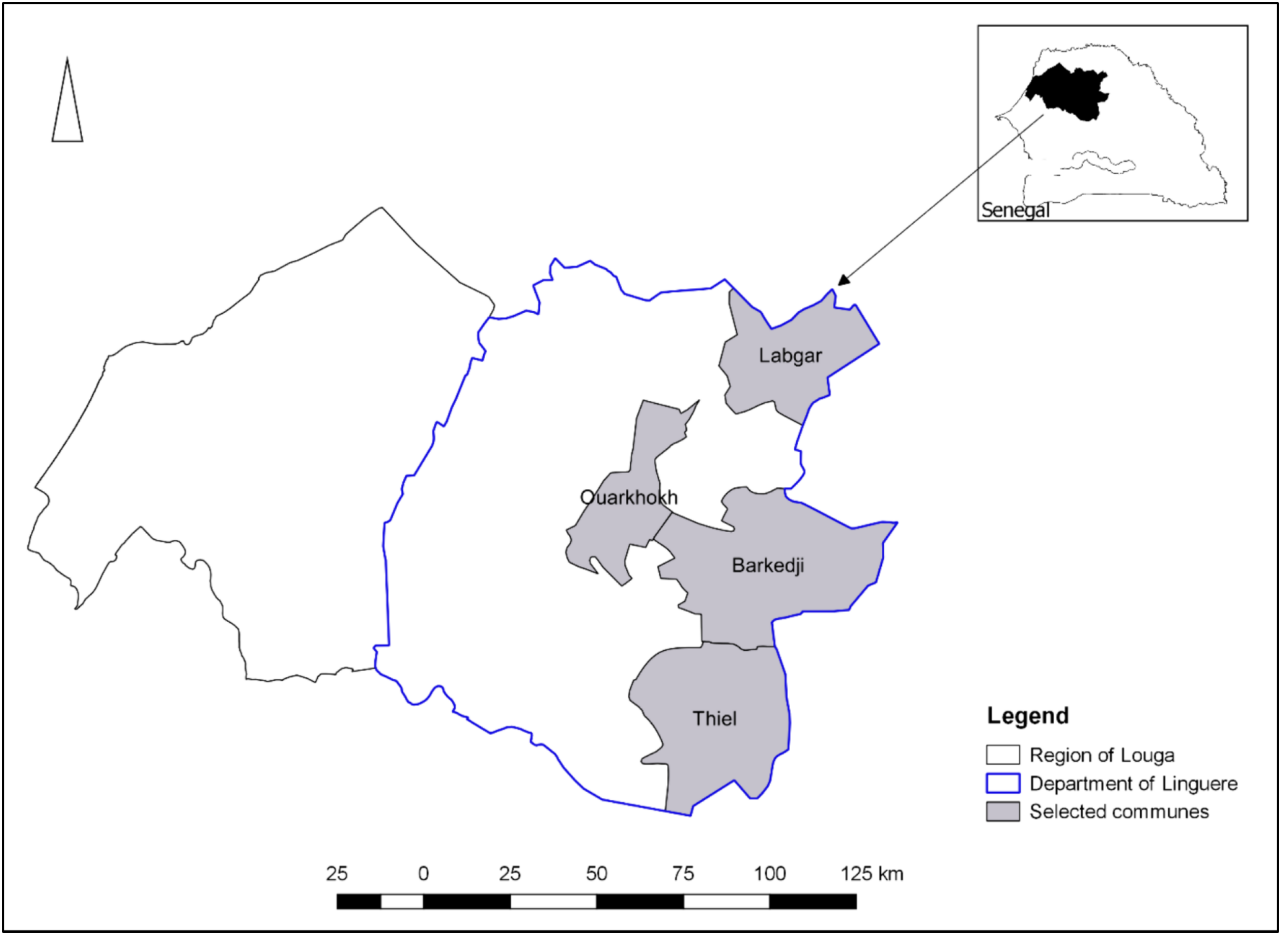
Study area mapping.

A multi-stage sampling including both probability and non-probability methods was used to select the SR producers. We purposively selected four communes, namely Labgar, Ouarkhokh, Barkedji, and Thiel, as the study sites to maximize the variation of parameters (Figure 1). Then, we randomly sampled the villages within each commune and the SR producers within each village, respectively, based on the list of the villages and the SR producers.

The sample size calculation was based on the number of SR producers in the study area and the proportion of the SR producers who were likely willing to vaccinate their SR against PPR. Indeed, based on the data of the national institute of statistic and demography in 2013 and 2022 (19, 20), the estimated number of the SR producers in 2022 were 2,531 in Labgar, 6,094 in Ouarkhokh, 6,674 in Barkedji, and 5,165 in Thiel. This makes a total of 20,464 SR keepers in the 4 selected communes combined.

We assumed that before the SR producers were willing to vaccinate or pay for vaccination, they would have detained a minimum of information (knowledge) about the disease. Several studies stated that SR producer knowledge enhancing on PPR disease and vaccination through sensitization campaigns lead to improve the participation of producers to vaccination (5, 21–24). Based on previous studies (23, 25), if the true prevalence for a parameter is 85% (representing the estimated proportion of the SR producers who had already heard about PPR), a confidence interval (CI) of 95% with 5% precision involves selecting a sample size of 196 respondents. Therefore, we sampled *n* = 200 SR producers for this study. To ensure the representation of villages, we randomly sampled five villages within each commune using the official list of villages. In each selected village, we compiled a list of all SR producers with the support of village leaders, from which we randomly sampled 10 producers, making a total of 50 producers per commune.

### 2.3 Questionnaire construction and administration

Data were collected by four trained enumerators from June 15 to 30, 2022. The data collection process involved conducting individual face-to-face interviews with the respondents using the Open Data Kit (ODK) application (26) by phone. Verbal consent to participate was obtained before each interview. The interviews were only conducted with consenting households. The selected households who refused to be interviewed were replaced. We used a questionnaire and a set of illustrative cards to implementation the choice experiment with the producers. The questionnaire was designed into three sections including socio-economic information, PPR disease (knowledge, occurrence, and vaccination) and WTV and WTP for vaccination questions. All respondents were at least 18 years old.

The vaccination options for the choice experiment were determined based on previous studies (7, 8, 23) and expert opinions. These experts had good knowledge on the PPR national eradication program and had previously conducted studies on PPR in Senegal. Six key vaccination attributes were identified: vaccination site (at home versus vaccination at the park), type of vaccine (thermolabile versus thermotolerant), vaccination strategy (group vaccination versus individual herd vaccination), PPR vaccination combined with additional services (PPR + other diseases versus PPR + deworming), and cost of PPR vaccination, which was divided into four levels XOF 75 (USD 0.12), 100 (USD 0.16), 150 (USD 0.24), 200 (USD 0.32), and XOF 250 (USD 0.41) (see Table 1). In terms of cost level, based on the existing cost, which was XOF 50, we added a lump amount to each level of cost (XOF 25 or XOF 50) considering the expert inputs to get four cost levels.

**Table 1:** Vaccination attributes and levels.

Attributes of vaccination	Option1	Option2	Option3
Vaccination site (VACSITE)	Vaccination in the park	Home vaccination	No choice
Type of vaccine (VACTYPE)	Thermotolerant vaccine	Thermolabile vaccine	
Vaccination strategy (VACSTRAT)	Group vaccination	Individual herd vaccination	
PPR vaccine + other services jointly offered (OSERV)	PPR vaccine + Deworming	PPR vaccine + another vaccine	
Nature vaccination (NATVAC)	Compulsory vaccination	Elective vaccination	
Cost of vaccination (PRICE)	Options: XOF 75 (USD 0.12), 100 (USD 0.16), 150 (USD 0.24), 200 (USD 0.32), and XOF 250 (USD 0.41)		

1CFA = 0.00164 USD, December 2023.

**Figure 2:**
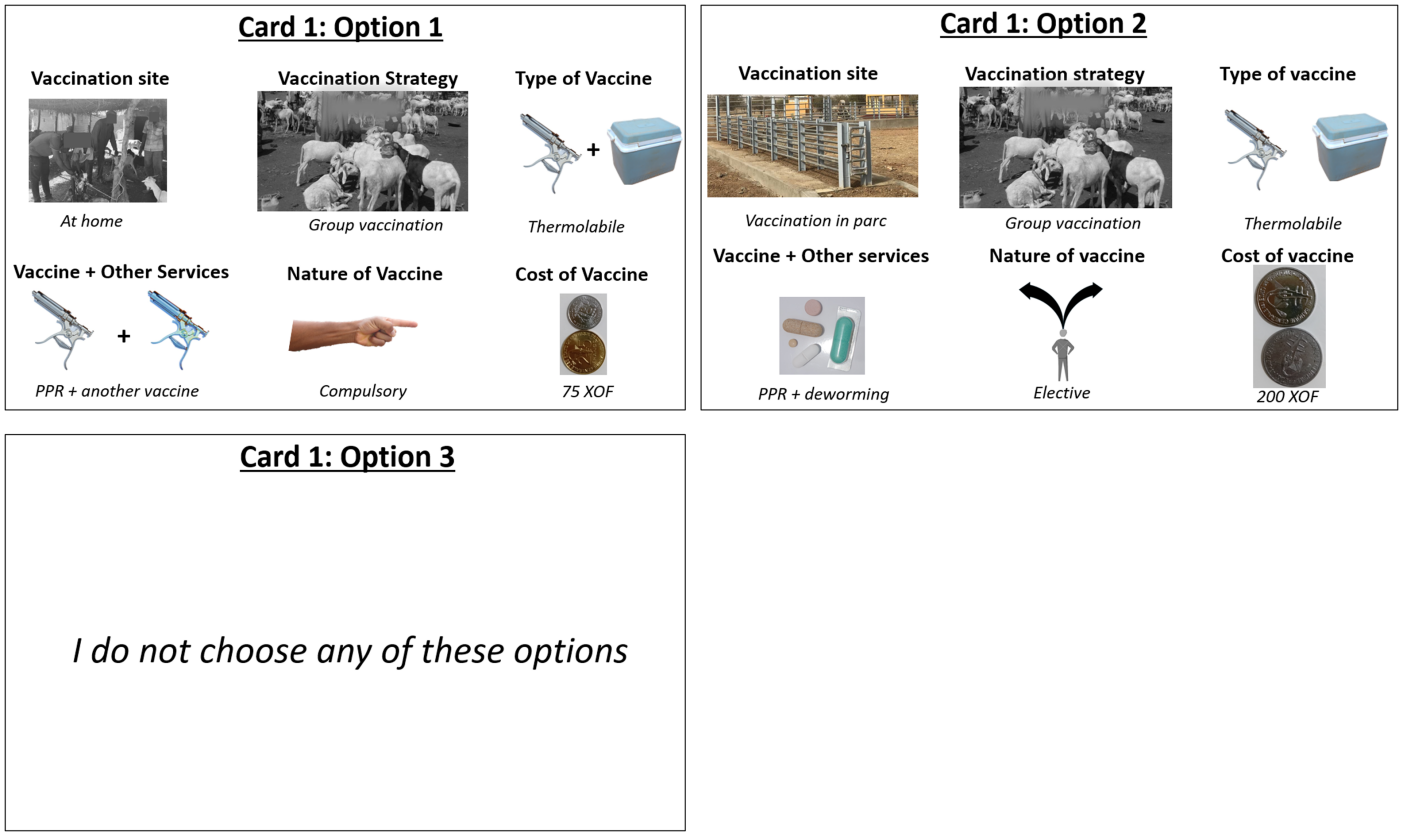
Example of a choice set options presented to producers.

To present the set of choices to producers responding to the survey, we constructed a choice of cards containing pictorial profiles that depicted the differences in vaccine attributes and levels. An example of a choice set options presented to producers is presented in Figure 2. Before the choice set experiment implementation, producers were provided information about the PPR disease, including its impact on the SR herds. The vaccination attributes and the way producers could make choices from the displayed cards were also explained. The cards were developed from the information in Table 1 as pictorial profiles. The producers were shown two vaccine choice options and a third option with a “no choice” option at a time for each of the eight combinations. The “no option” served as the status quo for the respondent who selects neither of the vaccine options that were presented to them. They were then asked to choose the preferred vaccine option they would purchase.

### 2.4 Choice experiment modeling

For the vaccine, there are five attributes each of which is presented at two levels, and a sixth attribute (price) at four levels. Combing the different attributes at different levels would yield a total of 128 product profiles for comparison which would make rating by respondents rather difficult. To reduce the number of product profiles to a more manageable number, we used an orthogonal fractional factorial design (27, 28) to generate 16 conjoint experiment profiles. The orthogonal profiles have the desirable property that there is no correlation between them. From the set of orthogonal profiles, 8 choice sets were generated as described in Aizaki and Nashimura (29) and Aizaki (30) which were presented to a total of 200 livestock keepers for rating during the survey. Each question presented three alternative options, including two product profiles, along with a third option of “none of the two options.” The dependent variable in the conditional logit (clogit) model captures the respondents’ preference for a given product profile. In contrast, the independent variables represent the attribute levels of the different product profiles presented during the interviews. For the “none of the two options” category (option 3), the attribute levels are entered as zeros, following the approach by Hideo and Nashimura (29). Consequently, the data consists of 4,800 rows, equivalent to 200 livestock keepers multiplied by eight choice questions and three alternatives per question.

The coding of data in the clogit model is crucial, as it influences the magnitudes of coefficients and their interpretation. Apart from price, the other five explanatory variables representing the attributes of the PPR vaccine are categorical, each with two levels (as indicated in Table 1). There are two commonly used methods for coding attribute levels in clogit models: effects coding and dummy-variable coding (15). In each of the two coding approaches, one level of each attribute must be omitted. In both effects coding and dummy-variable coding, each non-omitted attribute level is assigned a value of 1 when that level is present in the corresponding profile and 0 when another non-omitted level is present in the corresponding profile. The difference between the two coding methods is related to the coding of the non-omitted levels when the omitted level is present in the profile. With effects coding, all non-omitted levels are coded as −1 when the omitted level is present. With dummy-variable coding, all non-omitted levels are coded as 0 when the omitted level is present. In this analysis, the attribute levels are coded as dummy variables, taking a value of 1 if the level is present in the corresponding profile and 0 otherwise.

We use a c-logit model to estimate the WTP for various attributes by small ruminant producers when receiving a PPR vaccination service. One disadvantage associated with the c-logit model is that it suffers from independence of irrelevant alternative (IIA) assumption, forcing some researchers to turn to alternative models such as the nested logit and mixed logit models which are however computationally less tractable. Using immigration data from different regions in the U.S, an investigation of the 3 models by Christiadi and Brian (31) however found that their parameter estimates were of the same sign and were generally comparable in terms of statistical significance. It is for these reasons that the c-logit was used in this study.

The c-logit model is based on the random utility theory where a respondent’s utility (U) is divided into two components, that is, a systematic component (V) and a random component (e) (Equation 1) (29).


(1) U=V+e

As in Hideo and Nashimura (29) the systematic component of utility for “I do not choose any of these choices” is normalized to be zero while that of the other two alternative types of PPR vaccination services in each choice set can be represented as follows (Equation 2):


(2) $$V_{j}=ASC+\overset{5}{\underset{i=1}{\sum }}\beta_{i}X_{ij}+\beta_{p}P_{j}+\overset{5}{\underset{m=1}{\sum }}\beta_{m}C_{m}P_{j}$$

Where $V_{j}$ is the systematic component of utility derived from using alternative j of the two types PPR vaccination product profiles presented to a respondent in each choice set; ASC is the alternative specific constant; $X_{ij}$ is the level i^th^ attribute in alternative j; $P_{j}$ is the price level in alternative j; and $C_{m}$ is the respondent specific characteristic m; $\beta_{i}$ is the coefficient for attribute $X_{i}$ ; $\beta_{p}$ is the coefficient for price; and $\beta_{m}$ is the coefficient for the interaction between price and respondent specific characteristic m.

The respondent specific characteristics in the model included dummies on gender, level of education of household head together with geographical location of farms (commune) consistent with available literature on adoption of improved technologies by producers (30, 32). During the selection of respondent specific characteristics, joint inclusion of highly corelated variables (partial correlation coefficient > 0.7) was avoided as it would lead to the problem of multicollinearity and thus biased model estimates. The systematic component of the c-logit model in Equation 3 can be written as:


(3) $$\begin{array}{l} V_{j}=ASC+\beta_{1}\mathrm{VACSITE}+\beta_{2}\mathrm{VACTYPE}+\beta_{3}\mathrm{VACSTRAT}\\ \;+\;\beta_{4}\mathrm{OSERV}+\beta_{5}\mathrm{NATVAC}+\lambda_{p}\mathrm{PRICE}+\gamma_{1}\mathrm{PRICE}:GHH\\ \;+\;\gamma_{2}\mathrm{PRICE}:\mathrm{EDUC}+\gamma_{3}\mathrm{PRICE}:\mathrm{BARK}\\ \;+\;\gamma_{4}\mathrm{PRICE}:\mathrm{LABG}+\gamma_{4}\mathrm{PRICE}:\mathrm{QUARK} \end{array}$$

Where the variables for the different attributes of the vaccination service profiles are as defined in Table 1, GHH is gender of household head (1 if male and 0 otherwise), EDUC represent level of education of respondent (1 if no formal education and 0 otherwise), BARK represents Barkedji commune (1 if yes and 0 otherwise), LABG represents Labgar commune (1 if yes and 0 otherwise) and QUARK represents Ouarkhoh commune (1 if yes and 0 otherwise).

Note that in a c-logit model the values of the coefficients for attributes ( $\beta_{i})$ (also referred to as preference weights) show the relative preference of the varying levels of attributes by the consumers (15). The model was estimated using the c-logit function contained in the survival package in the R-computer package (33). To measure the goodness of fit of the clogit model, the likelihood ratio test (34) was used. The loglikelihood ratio is −2 times the difference between the log likelihoods of the non-restricted and the restricted model. As observed by Aizaki (35) and Berendsen (36), the marginal willingness to pay (WTP) for a discrete change in the level of an attribute is calculated using the formula in Equation 4:


(4) $$WTP_{i}=-\left(\frac{\beta_{i}}{\beta_{p}}\right)$$

Where $WTP_{i}$ is the WTP for discrete change in the level of the attribute. Higher levels of WTP imply higher relative importance of the respective attribute.

### 2.5 Descriptive statistics

All frequency calculation were made by using this formula in Equation 5:


(5) $$f_{i}=\frac{n_{i}}{N}*100$$

Where f_i_ is the frequency, A the n_i_ the value of the items and, *N* the sample size.

For all frequencies calculated, we estimated the confidence interval (CI) at 95% using this formula in Equation 6:


(6) $$CI=\bar{x\;}\underline{+}\;z\frac{S}{\sqrt{n}}$$

Where CI is the Confidence Intervale, x̄ the population mean, z the confidence level value, S the sample standard deviation and, *n* the sample size.

## 3 Results

### 3.1 Socio-economic information and knowledge of producers about peste des petits ruminants

**Figure 3:**
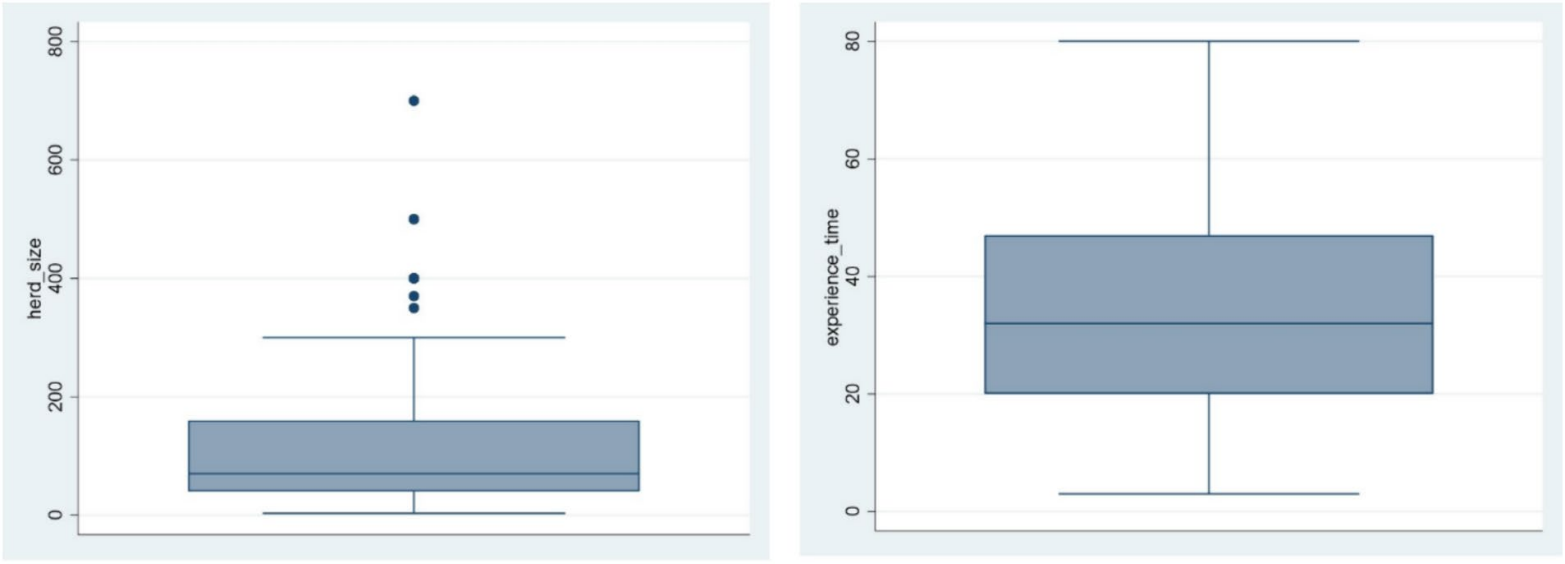
Distribution of herd size and number of years in small ruminants keeping.

The study revealed several key socio-economic characteristics and knowledge levels of producers regarding PPR among the respondents. Most of the participants were male (68%) and had an average age of around 45 years. An important proportion of the respondents (62.5%) reported having no formal education. Livestock, particularly SR, were the primary source of income for 85% of the respondents. Additionally, 61% of them stated that they were co-owning the SR herds with someone else. The survey also indicated that sheep were kept more commonly than goats, with 77.5% of respondents predominantly keeping sheep. The size of the SR herd ranged from three to 700 heads, with a median herd size of 118 heads. Notably, the respondents had an average of 33 years of experience in SR keeping (Table 2; Figure 3).

**Table 2:** Summary statistics for participants variables.

Items	Frequency (*n* = 200)	Percentage	Percentage at 95% CI (%)
Gender			
Male	168	84	79–89
Female	32	16	11–21
Education			
No level	125	62	56–69
Literacy	41	20	15–26
Primary	16	8	4–12
Secondary and beyond	18	10	6–14
Livestock as the main source of income			
Yes	171	85	81–90
No	29	15	10–19
Small ruminant type number in the herd			
More sheep	155	77	72–83
More goat	19	10	5–14
No notifiable difference	26	13	8–18
Role in the small ruminant herd keeping			
Owner alone	75	37	30–44
Co-owner with someone else	124	61	54–68
Other (entrusted or shepherd)	3	2	0–4

An important proportion of the respondents (72%) reported that they could identify symptoms of PPR. The most frequently observed symptoms attributed to PPR they had recalled having occurred within the past 12 months to the survey were diarrhea and/or cough, discharge from the nose, tearing, oral erosions, and fever, with prevalence rates of 21, 20, 19, 15, and 11%, respectively. Moreover, in 65% of cases, the clinical signs observed by the respondents aligned to the syndrome of PPR within the past year, with death occurring in 45% of the cases. However, in 42% of the cases, sick animals were reported to have recovered. Surprisingly, most producers (80%) reported that they had never received any sensitization or awareness programs about PPR. Additionally, in 81% of the cases, the decision to vaccinate against PPR was made by the owner of the herd (Table 3).

**Table 3:** Various information on PPR occurrence in the herd.

Items	Frequency (*n* = 200)	Percentage	Percentage at 95% CI (%)
Capability to identify PPR symptoms			
Yes	144	72	66–78
No	56	28	22–34
Main symptoms observed in the herd within the 12 past months			
Fever	63	11	7–16
Diarrhea and/or cough	118	21	16–27
Tearing	107	19	14–25
Discharge from the nose	112	20	15–26
Oral erosions	87	16	11–21
Conjunctivitis	37	7	3–10
No symptom observed	31	5	2–9
PPR syndrome observed within the 12 past months			
Yes	130	65	58–72
No	70	35	28–42
Event happened during PPR outbreak in the herd			
Sick animals cured	102	42	35–49
Sick animals were sold immediately	17	7	3–11
Sick animals died	110	45	39–52
Sick animals were slaughtered	10	4	1–7
Others	3	1	0–3
Respondent received a sensitization on PPR			
Yes	40	20	14–26
No	160	80	74–86
PPR sensitizer			
Government animal health worker	22	48	41–55
Private animal health worker	1	2	0–4
Community worker	18	39	32–46
Relative or another producer	5	11	7–15
PPR sensitization canal			
Community radio	11	25	20–32
Sensitizing meeting	21	49	42–56
Other	11	25	20–32
Decision maker for PPR vaccination			
Herd owner	166	81	13–14
Relative	22	11	13–14
Herd keeper	4	2	1–5
Other	12	6	3–10

**Figure 4:**
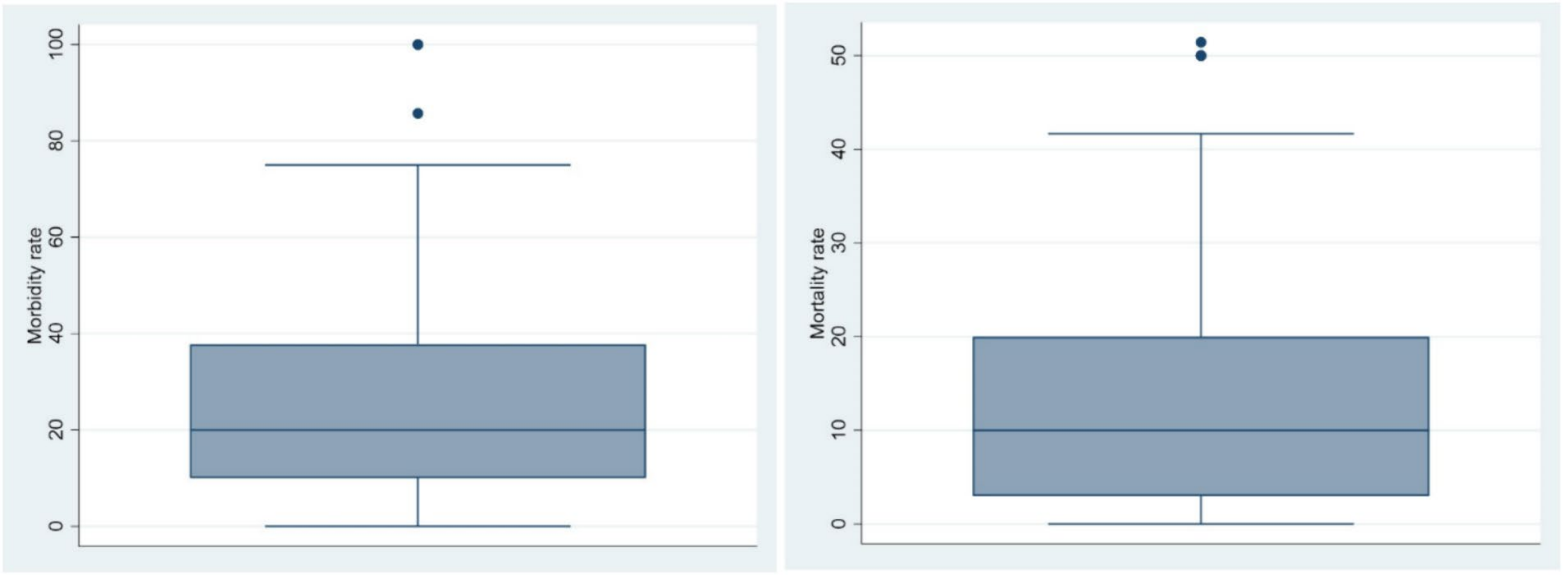
Distribution of estimated PPR morbidity and mortality rate reported by the respondents.

According to the respondent estimations, the morbidity rate (the number of sick animal divided by the number of animal in the herd) and mortality rate (the number of died animal from PPR divided by the number of animal in the herd) of PPR were approximately 20 and 10%, respectively, with the past 12 months (Figure 4).

### 3.2 Factors associated with willingness to vaccinate the PPR vaccination

Table 4 illustrates that 39% of the respondents are yet to vaccinate their herds against PPR. The primary reasons provided for not vaccinating were lack of awareness about PPR vaccination (51%), unavailability of the vaccine (29%), and lack of trust in the efficacy of vaccination (10%). In terms of vaccination preferences, a majority of the respondents (68%) expressed a preference for repetitive annual vaccination, while only 23% favored a one-off vaccination that conferred lifelong immunity (Table 4).

**Table 4:** Socioeconomic factors that influence the decision to vaccinate.

Items	Frequency (*n* = 200)	Percentage	Percentage at 95% CI (%)
Already vaccinate the herd against PPR			
Yes	122	61	54–68
No	78	39	32–46
Raison for non-vaccination			
I have not heard of the PPR vaccination	62	51	44–58
I heard about vaccination, but the vaccine was not available to me	35	29	23–35
I believe that vaccination has no benefit for my herd	1	1	0–2
I believe that vaccination could cause problems for my herd	3	2	0–5
The cost of vaccination is expensive	1	1	0–2
The traceability of vaccinated animals is too difficult	1	1	0–2
The current vaccination system does not suit me	2	2	0–3
The current vaccination period does not suit me (transhumance, wintering, etc.)	3	2	0–5
I do not believe in it for cultural reasons	13	11	6–15
Preferred frequency for PPR vaccination			
Every year	128	64	57–71
Every 2 years	14	7	4–12
Every 3 years	6	3	1–7
Lifetime immunity	47	23	18–29
Other	5	2	0–5
Preferred strategy for PPR vaccination			
Travel to the vaccinator in another locality	16	8	5–13
The vaccinator travels to my locality	182	91	87–95
Other	2	1	0–2
Preferred animal marking type			
Paint	99	49	43–56
Ear notch	15	7	4–11
Ear tag	73	37	30–43
None	3	1	0–3
Other	10	5	2–8
Preferred vaccinator type			
Government vaccinator	122	61	54–68
Private vaccinator	5	2	0–5
Community vaccinator	38	19	14–25
Whatever	33	17	12–22
Other	2	1	0–2
Preferred payment method for PPR vaccination			
Payment by cash	193	96	94–99
Payment by credit	6	3	1–5
Payment in kind	1	1	0–1
Opinion on the mandatory vaccination			
Good idea	175	87	83–92
Bad idea	18	9	5–13
Do not know	7	4	3–11
Will you tend to vaccinate more if the vaccination includes deworming?			
Yes	198	98	11–15
No	1	1	0–1
Do not know	1	1	0–1
Will you tend to vaccinate more if the vaccination includes another vaccine			
Yes	195	97	95–100
No	4	2	0–4
Do not know	1	1	0–1

**Figure 5:**
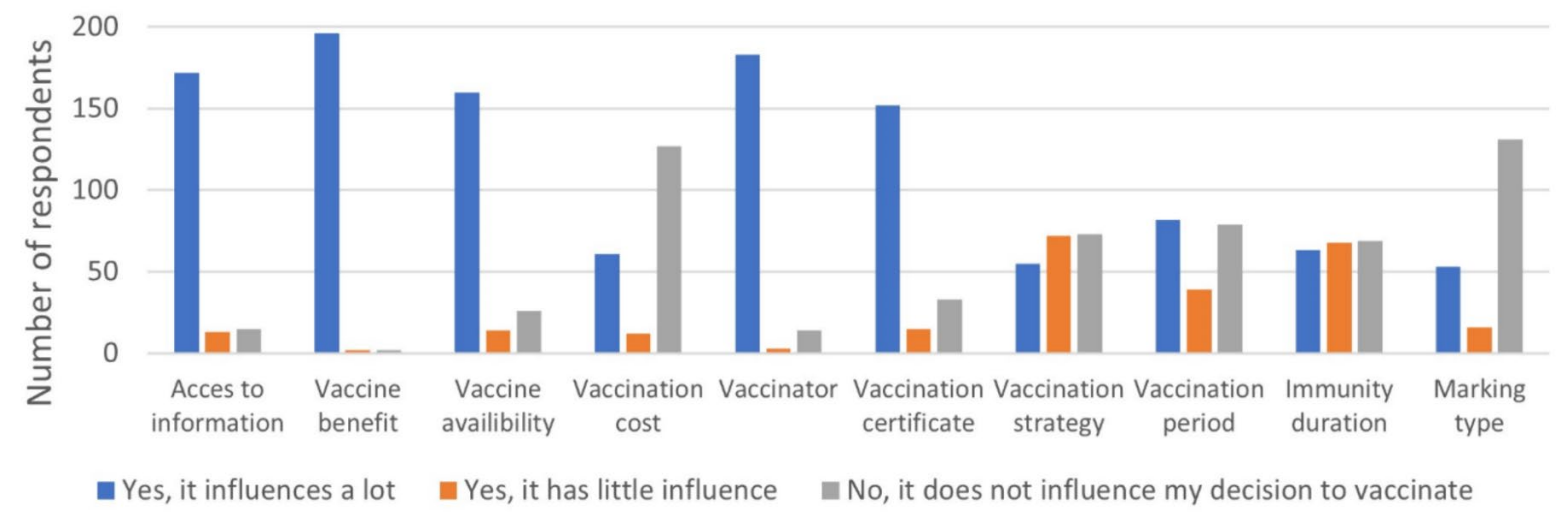
Influence of various factors on the decision to vaccinate.

The producers reported that several factors influenced their decision to vaccinate. The most influential factors were the perceived benefits of vaccination (98%), the type of vaccinator (91%), access to information (86%), availability of the vaccine (80%), and possession of a vaccination certificate (76%) (Figure 5).

### 3.3 Willingness to pay for vaccination

During the questionnaire administration, the response rate was 100%. All 200 producers participated to the survey and answered all survey questions.

Table 5 displays the results of the c-logit model and provides estimates of the WTP for different attributes. The loglikelihood test confirms that the model is statistically significant at a 1% level, indicating its reliability. In a c-logit model, the coefficient values represent the relative preference of consumers for the various levels of attributes (15). Using dummy-variable coding, the *p*-values indicate the statistical significance of the difference between the estimated preference weight and the reference category.

**Table 5:** Results of the clogit model together with estimates of WTP for the various attributes of the PPR by livestock keepers.

Variable	Description	Coef	se (coef)	[95% Conf. Interval]		Marginal WTP
Alternative specific constant	Alternative specific constant	1.96	7.09	−11.94	15.86	
Home vaccination***	Vaccination site (0 = Parc; 1 = Home)	0.74	2.09	−3.36	4.84	−183.53
Thermotolerant vaccine	Type of vaccine (0 = Thermolabile; 1 = Thermotolerant)	−0.19	0.83	−1.82	1.45	46.93
Individual herd vaccination strategy***	Vaccination strategy (0 = Group vaccination; 1 = Individual herd)	0.45	1.57	−2.63	3.53	−112.85
PPR vaccination + Other services jointly offered	Other services jointly offered (1 = Other vaccine; 2 = Deworming; 0 = None)	0.03	1.03	−1.99	2.05	−7.17
Elective vaccination***	Nature vaccination (0 = compulsory; 1 = Elective)	0.33	1.39	−2.39	3.05	−81.98
Cost of vaccination***	Cost of vaccination (75, 100, 150, 200, 250 FCFA)	−0.004	1.00	−1.96	1.96	1.00

Likelihood ratio test = 866.9 on 12 df, *p* = < 2.2e−16 *n* = 4,800, number of events = 1,600. ***, **, and * indicates significance at 1, 5 and 10% level, respectively.

In our analysis, several levels of vaccination service profiles were found to be statistically significant. These include the preference for vaccinating animals at home compared to vaccination at the park (preference weight = 0.74, *p* = 0.01), the preference for an individual herd vaccination strategy over group vaccination (preference weight = 0.45, *p* = 0.01), the preference for elective participation during vaccination campaigns by producers rather than compulsory participation (preference weight = 0.33, *p* = 0.01), and the cost of the vaccination service (preference weight = −0.004, *p* = 0.01). The negative coefficient for the cost of vaccination service indicates that livestock producers prefer lower-cost vaccination services.

On the other hand, the preference weights for using a thermotolerant vaccine instead of a thermolabile vaccine and for a PPR vaccination service which includes vaccination combination with another disease compared to deworming as a combined service were not statistically significant (Table 5).

## 4 Discussion

### 4.1 Farmer knowledge about peste des petits ruminants disease

The findings of the study indicate that most producers (two-thirds) seem to possess knowledge about PPR, despite receiving limited awareness about the disease from veterinary services. PPR has been recognized as an old disease in the West African sub-region, with its discovery dating back to 1942 in Côte d’Ivoire (37) and reported since 1955 in Senegal (38). SR producers, having accumulated over 30 years of experience in livestock farming, have obviously gathered experience that enable them to identify suspicious cases of PPR through the symptoms (39). The symptoms reported by producers, such as sudden fever, eye and nose discharge, respiratory issues, diarrhea, and mortality, align well with the disease, although laboratory confirmation is the ideal option to confirm PPR in a herd.

Our findings further underscore the persistence of PPR in these communities, despite vaccination efforts. However, producers have reported relatively lower morbidity and mortality rates of around 20 and 10%, respectively, compared to the higher rates typically associated with PPR (ranging from 70 to 100%) (40). These findings shed light on the respondents’ knowledge and experiences with PPR, highlighting the need for targeted sensitization campaigns and owner-led vaccination initiatives to mitigate the impact of the disease on small ruminant populations.

### 4.2 Factors associated with willingness to vaccinate against PPR

The findings indicate that approximately 40% of producers reported never having vaccinated their herds against PPR, citing reasons such as a lack of awareness about PPR vaccination and unavailability of the vaccine. These results align with the relatively low national coverage rate observed at the national level despite the efforts of government services and their partners (1). The challenges related to vaccine supply and the seasonal movement of herds during transhumance (1, 5, 18), as highlighted in previous studies, likely contribute to this situation. Additionally, only 20% of the respondents stated that they had received awareness about the disease from veterinary services. This emphasizes the critical need to strengthen community-based information and awareness activities, not only regarding PPR but also focusing on preventive measures, including vaccination.

When asked about their preferred frequency of vaccination, most producers expressed a preference for annual vaccination (repeated vaccination), while only 23% indicated a preference for a single vaccination that provides lifelong immunity. It is common for producers to adhere to the practice of annual vaccination for diseases such as Bovine Contagious Peri-pneumonia (CBPP), pasteurellosis, and blackleg (1). This preference for frequent vaccination may stem from the belief that more frequent vaccination offers better protection for animals. In a study conducted by Ledaprier et al. (5), in the Ferlo region of Senegal (where our study site is located), nearly all producers emphasized the importance of annual PPR vaccination. Balde et al. (18) also noted that producers have integrated annual booster vaccinations into their routine and consider them necessary. They believe that the potential savings from a single vaccination cannot justify the associated risks and negative consequences, such as decreased confidence in vaccination or potential implications for other diseases. However, in the global eradication strategy, the current PPR vaccine provides lifelong immunity and requires a single vaccination (4, 41). It is important to raise awareness among producers about the benefits and effectiveness of a single vaccine dose that confers lifelong immunity. This will also help to avoid vaccine wastage.

According to the statement of the producers, several factors can influence their decision to vaccinate, including the perceived benefits of vaccination, the type of vaccinator, the access to information about vaccination, the nature of the vaccine itself, as well as availability of a vaccination certificate. The importance placed on the benefit of vaccination highlights the need for producers to be convinced of the positive outcomes associated with vaccinating their animals, such as the efficacy and safety of the vaccine. The role of the vaccinator is also crucial, as it involves a matter of trust. Balde et al. (18) observed in the Ferlo area in Senegal that some producers preferred to have their animals vaccinated only by vaccinators who were their relatives or who are known to them. In cases where their preferred vaccinators were not part of the vaccination team, some producers may refuse to vaccinate. In some instances, producers may even take the initiative to purchase the PPR vaccine themselves and vaccinate their animals (18).

The producers also emphasized the significance of vaccination certificates as a motivating factor for vaccination. These certificates serve as written proof that the herd has been vaccinated against a specific disease and can facilitate transhumance movements with veterinary posts. This aspect may encourage producers to vaccinate more, even though the certificate does not indicate an obligation to vaccinate each individual animal, as it is issued for the entire herd (5). In the context of the eradication process, it could be considered to introduce vaccination certificates that differentiate between partially and fully vaccinated herds and restrict the movements of the herd, accordingly, aiming to vaccinate nearly all the animals.

These findings emphasize the importance of raising awareness about PPR vaccination, ensuring vaccine availability, and providing accessible and reliable information to producers.

### 4.3 Willingness to pay for vaccination

The results of the conjoint analysis indicate that four vaccination attributes have a significant impact: vaccination site, vaccination strategy, nature of vaccine, and cost of vaccination.

#### 4.3.1 Home versus park vaccination

The vaccination site has emerged as an important attribute, with producers expressing a preference for home vaccination. This preference may be motivated by a desire to minimize additional costs associated with moving the herds to a distant place and to reduce the risk of disease transmission between herds. A study conducted in Burkina Faso revealed that most vaccination sessions are conducted at the homes of the producer (42). Although door-to-door vaccination can be expensive and labor-intensive for vaccinators, it facilitates animal restraining, reduces the risk of disease spread and improves vaccination adherence because of convenience (42).

#### 4.3.2 Individual herd versus group vaccination

Additionally, producer indicated a preference for individual herd vaccination over group vaccination, which involves mixing herds. Although door-to-door vaccination can be expensive and labor-intensive for vaccinators, it facilitates animal restraint, reduces the risk of disease spread, such as PPR, and improves vaccination coverage (42, 43).

#### 4.3.3 Elective versus mandatory vaccination

The producers also expressed a preference for elective vaccination rather than mandatory vaccination. Elective vaccination involves imposing sanctions to producers who refuse to vaccinate their herd. Sanctions may include restrictions on animal movement for grazing or watering, banning access to livestock markets, or other measures implemented by authorities. However, these measures may not be suitable for all producers, as some may prefer the freedom to choose whether to vaccinate their animals. Moreover, a mandatory vaccination does not imply necessarily free-cost vaccination. Some producers do not perceive the benefits of vaccination, while others consider the cost to be high and therefore resist mandatory vaccination. Thus, imposing the vaccination without a large awareness campaign with the threat of various sanctions could mean forcing them to pay for something they are not persuaded of. In recall, the vaccination cost is one of the key factors that impact the producer’s willingness to pay for PPR vaccination. Ledrapier et al. (5) found in their study that only 52% of producers would vaccinate their herds if vaccination were compulsory, underscoring the importance of promoting understanding and acceptance of vaccination beyond its mandatory nature.

#### 4.3.4 Limitation of the method used

It should also be noted that much more improved methodologies for estimation of WTP have been developed including use of Hierarchical Bayesian (HB) mixed logit models, but these often tend to be less computationally tractable (31, 44). The HB model enables estimation of individual respondent’s level of WTP as opposed to the c-logit estimation which gives an estimate of the average level of WTP in a population. The HB method yields detailed information that is useful when studying developed markets characterized by some sophistication where segmentation might be a key product promotion strategy (44). On the other hand, payment for vaccination against PPR among livestock producers in Senegal is a market that is in its emergence stages and the employment of a c-logit model was deemed sufficient in informing the general direction that vaccination strategies should take. However, as the PPR vaccine market develops and becomes more complex, future studies will need to explore the potential for segmentation as a strategy for enhancing vaccination coverage by employing more rigorous WTP analysis approaches such as the HB estimation.

## 5 Conclusion

This study aimed to assess the willingness of small ruminant producers to vaccinate their livestock against PPR, a highly lethal disease affecting SR in Senegal and other regions where SRs are raised. The findings revealed that while producers are generally aware of PPR, a significant proportion of them lack information about PPR vaccination. Various factors contributed to their limited participation in vaccination programs, such as lack of trust about the current vaccination strategy, which involves a single dose for lifelong immunity, raising concerns about its effectiveness and benefits.

To address this issue, it is crucial to educate producers about the importance of adhering to the current vaccination schedule and the benefits of vaccination. Additionally, efforts should be made to overcome logistical challenges that hinder producers from accessing vaccines. Extension systems and veterinary services are vital in delivering the necessary knowledge and support in this regard.

In summary, the study highlights the need for enhanced education and awareness regarding PPR vaccination among SR producers. Alongside this, logistical barriers must be addressed to improve the participation of producers in vaccination programs. The involvement of extension systems and veterinary services is essential to achieve these objectives.

## Data availability statement

The original contributions presented in the study are included in the article/supplementary material, further inquiries can be directed to the corresponding author.

## Ethics statement

The requirement of ethical approval was waived by Service départemental de l’Elevage et des productions animale de Linguère - Departemental service of livestock of Linguere for the studies involving humans because the study was implemented with relevant local government in charge of disease control in the area (Service départemental de l’Elevage et des productions animale de Linguère - Departemental service of livestock of Linguere). We obtained a letter of support from them before we start the study. The studies were conducted in accordance with the local legislation and institutional requirements. The ethics committee/institutional review board also waived the requirement of written informed consent for participation from the participants or the participants’ legal guardians/next of kin because most producers were illiterate and were not able or used to signing consent. Thus the project team decided in accordance with local partners to ask the oral consent. This is a common approach in the area.

## Author contributions

GI: Writing – review & editing, Writing – original draft, Visualization, Methodology, Investigation, Formal analysis, Data curation, Conceptualization. FW: Writing – review & editing, Writing – original draft, Visualization, Software, Methodology, Formal analysis, Conceptualization. SB: Writing – review & editing, Visualization, Validation, Supervision, Methodology. SS: Writing – original draft, Writing – review & editing, Methodology, Investigation, Conceptualization. CD: Writing – review & editing, Investigation, Data curation. PS: Writing – review & editing. ML: Writing – review & editing. MD: Writing – review & editing, Writing – original draft, Visualization, Validation, Supervision, Resources, Project administration, Methodology, Funding acquisition, Conceptualization.
